# A complicated true sliding hernia presenting as a spontaneous enteroscrotal fistula in an adult

**DOI:** 10.4103/0974-2700.58661

**Published:** 2010

**Authors:** Saravana Rajamanickam, Ashok Yadav, Anurag Rai, Devendra Singh, Abhinav Arun Sonkar

**Affiliations:** Department of Surgery, Chhatrapati Shahuji Maharaj Medical University (Formerly King George's Medical University), Lucknow, India

**Keywords:** Complications of inguinal hernia, entero-cutaneous fistula, scroto-fecal fistula

## Abstract

A 26-year-old man presented with an irreducible right inguino-scrotal swelling and fecal discharge from the scrotum. Exploratory laparotomy and inguinal exploration revealed that the caecum, appendix, and terminal ileum had herniated into the scrotum and had perforated through the skin forming a fecal fistula. The herniated gangrenous bowel was resected and a stoma fashioned. Spontaneous entero-scrotal fistulae are very rare and eight pediatric cases have been mentioned in literature till date. We report the first case of true sliding hernia presenting as spontaneous entero-scrotal fistula in an adult.

## INTRODUCTION

Inguinal herniae are quite common in surgical practice. However, sliding herniae are rare with an incidence of 2 to 5%.[1] The exact diagnosis of sliding hernia is made on the operating table. Hernia *per se* is not a difficult surgical condition but assumes importance in resource constrained countries like India where illiteracy and lack of finances lead to delay in seeking medical care, ultimately leading to increased morbidity. Complications like incarceration, strangulation, and obstruction of the herniae are hence not uncommon in surgical practice in such countries.

## CLINICAL DETAILS

**Figure 1:**
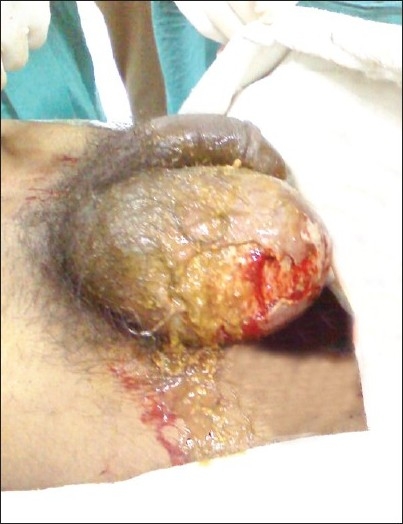
Fecal discharge from the right scrotum

**Figure 2:**
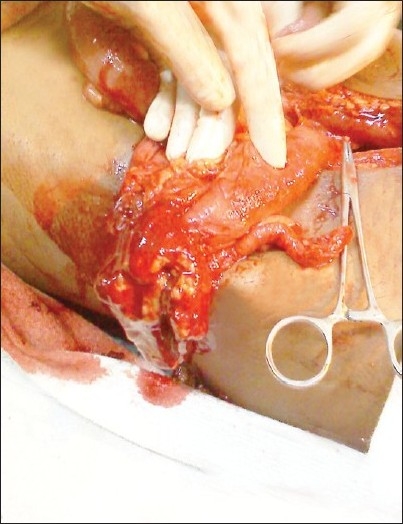
Perforated caecum and appendix

**Figure 3:**
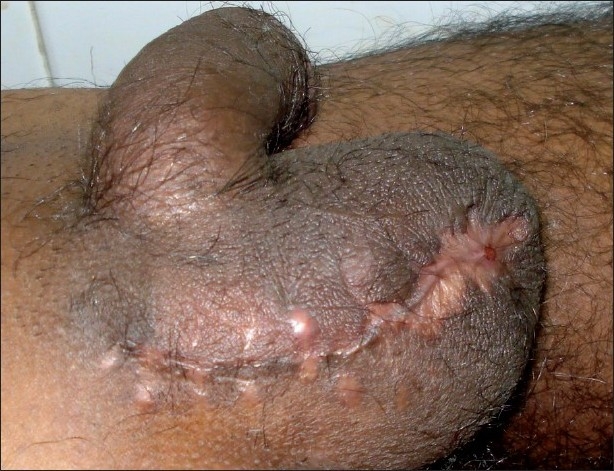
The scrotal wound on followup

**Figure 4:**
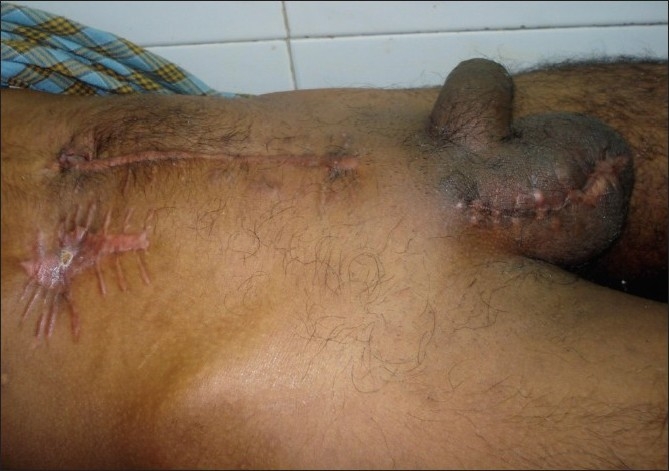
The patient at three months' followup

A 26-year-old farmer presented to the Emergency department with acute abdomen, fecal discharge from the right inguinoscrotal region and shock. The patient elaborated the presence of a reducible swelling involving the right ingunoscrotal region since 5 years that became painful and irreducible the week prior to admission. Gradually abdominal distension, pain, vomiting, and obstipation ensued. Foul smelling discharge emanated from the right scrotal region 3 days back. The patient was dehydrated, had a feeble pulse (112/min), and was tachypneic (26/min). His blood pressure was 70/52 mm Hg. Clinical examination of the abdomen revealed it to be grossly distended and tender. Diffuse guarding, rigidity, and a tympanic abdomen pointed towards peritonitis. The scrotal area was found to be stained with greenish material with fecal odor. A closer examination revealed fecal material flowing out of an opening at the root of the right scrotum, the surrounding skin being inflamed and macerated.[Figure 1] The inguinal hernia on the right side was tense, tender, irreducible and had a doughy consistency. The patient was immediately resuscitated with 2 liters of Ringer lactate with two large bore 16G catheters and reassessed in one hour when the patient's blood pressure improved to 106/74 mmHg. The patient was given 2g intravenous ceftriaxone, 500mg amikacin, and 500mg metronidazole. The patient's initial blood parameters showed hemoglobin of 8 g/dl, total leukocyte count 16500 cells/mm^3^, serum urea (48mg/dl), serum creatinine (0.8mg/dl), serum sodium, potassium 132 and 3.5 mEq/L, respectively. A plain abdominal radiograph of the abdomen revealed pneumoperitoneum. Further radiological investigations were limited to an ultrasound in the Emergency department showing free intraperitoneal air and fluid along with the confirmation of bowel loops in the inguinal hernia. Dye studies to identify the source of the fistula were withheld as the obstructed bowel contents in the strangulated inguinal hernia was the obvious source of the fecal fistula and the patient was planned for exploration. One unit of fresh whole blood was transfused prior to surgery. The patient underwent exploratory laparotomy through a vertical midline incision that revealed moderate amount of pus and dirty flakes contaminating the peritoneal cavity. The caecum with the appendix had traversed through right inguinal canal to lie in the scrotum. The caecum had perforated through the right scrotal skin with fecal discharge. The herniated bowel was gently maneuvered out of the scrotum and brought into the abdomen [Figure 2]. A resection of the caecum and terminal ileum was performed and an end ileostomy with colonic mucus fistula was fashioned. An anatomic repair of the hernia was performed and no prosthetic mesh used. The scrotal skin and adjoining tissue was debrided after carefully ensuring the viability of the adjoining right testis. The patient recovered without complications. The scrotal wound was dressed daily until healthy granulation allowed safe secondary approximating sutures. A reversal procedure for the stoma was done two months later. Postoperative followup pictures at three months are shown in Figures 3 and 4.

## DISCUSSION

Sliding hernia (synonym: hernia en glissade) is a result of slipping of the parietal peritoneum due to elongated mesentery.[1] In the right side caecum, appendix and ileum form the posterior wall of the hernia and in the left side the sigmoid colon.

## SEARCH STRATEGY

An English literature search was done in Pubmed/MEDLINE and Ovid databases using the keywords “enterocutaneous fistula,” “complications of inguinal hernia”, “scroto-fecal fistula,” and “strangulated hernia”. Enterocutaneous fistulae due to previous surgery and Crohn's disease were excluded from the articles generated.

## REVIEW OF LITERATURE

Till date most cases of inguinal herniae leading to scrotal fecal fistula have been described in the pediatric age group.[2–4] There has been only one report of inguinal hernia in an adult that led to a spontaneous scrotal fecal fistula. Our case is unique in that a true sliding hernia presented as a scrotal fecal fistula. Complication rates for inguinal hernia are in the range of 4 to 10% with less than 1% leading on to strangulation. Risk for strangulation is greatest at infancy. This seems to be due to the greater incidence of such herniae and the narrower deep inguinal ring in infancy predisposing to incarceration. There has been no report, however, on the percentage of incarcerated herniae that perforate. Gasper *et al.*[1] reported that left sided true sliding inguinal herniae were more frequent than the right. An early study by Ryan *et al.*[4] mentioned that left sided sliding inguinal herniae were 4.5 times more common than the right. However, there was no mention on the rates of strangulation of such sliding herniae. Ghritlaharey *et al.*[2] in their review of the 8 reported cases of entero-scrotal fistulae showed that seven were on the right side, similar to the findings in our patient, and only one on the left. Perforation and fistula formation from sliding inguinal herniae is still not reported in literature. The only reported spontaneous entero-scrotal fistula in an adult was due to perforation of a herniated segment of the ileum on the right side.[5] There has been a similar report of caecal and appendiceal perforation leading to a scrotal fistula albeit in a child.[6] In the previous reports, both the paediatric and of the lone adult, there was only one mortality in a 30-day-old infant. Right-sided herniae seemed to be more prone to strangulation and subsequent perforation shown from the eight cases that were on the right and only one case that presented on the left side. Two patients required orchidectomy due to gangrene. Complications include anastamosis leaks, scrotal wound infection, and septicemia.[2]

## PATHOPHYSIOLOGY OF THE SCROTAL FISTULA

Strangulated hernial contents perforate when the twisted and thinned out bowel gives way. The ensuing leaking bowel contents macerate surrounding tissue, suppurate through the fascial planes, and finally end up forming a fistulous communication with the skin. The skin of the inguino-scrotal skin being thin probably favors fistula formation.

## EMERGENCY MANAGEMENT

In such patients, immediate surgical management is warranted as the fecal contents could destroy large areas of both skin and surrounding tissue. The paediatric series of inguino-scrotal fistulae show that the testis on the side of the hernia was either non-viable or required removal in two cases. Large areas of devitalized skin and subcutaneous tissue need to be debrided and the adjoining testis checked for viability. The scrotal wound, in our patient, was left to heal by secondary intention as primary suturing was not appropriate. Daily wound care resulted in healthy granulation and secondary suturing was performed by mobilizing scrotal skin on the 6^th^ postoperative day. Greater skin loss would require skin grafting as a delayed procedure.

Differential diagnosis of the acute scrotum includes acute torsion of the testes, torsion of the testicular appendage, acute epididymo-orchitis and Fournier's gangrene. In most of the cases a thorough clinical examination easily clinches the diagnosis. Torsion of the testes presents as an extremely tender testicle. Pain not relieved by elevation of the affected testis, a normal urinalysis and blood count differentiate it from acute epididymo-orchitis. Doppler evaluation of the testicular vasculature might be required in equivocal cases. Torsion of the testicular appendage presents similar to torsion of the testis and is frequently discovered at surgical exploration. Fournier's gangrene is a severe polymicrobial infection of the scrotal skin with sparing of the testes. In the initial stages, the scrotal skin is extremely painful, edematous, and inflamed. In advanced cases, necrosis of the scrotal skin ensues shamefully exposing the testes. Patients with spontaneous enterocutaneous fistulae should be evaluated for Crohn's disease. Spontaneous enterocutaneous fistula in patients with tuberculosis of the bowel has been reported in literature in areas where the disease is endemic.[7]

Vigorous resuscitation to correct fluid and acid-base deficits is of prime importance. Exploration is inevitable in most of the cases ensuring correction of shock and electrolyte imbalances present. Difficulties during inguinal dissection must be anticipated. Care must be taken to identify the contents of the hernia, most importantly the bladder, to avoid inadvertent injury. General condition of the patient guides primary bowel anastamosis or fashioning a stoma. Increasing experience with laparoscopic surgery has led to reports of the laparoscopic management of enterocutaneous fistulae in the setting of Crohn's disease.[8] The feasibility of such an approach to entero-scrotal fistulae is far fetched at least for the time being.

## RADIOLOGY

Plain radiography of the abdomen is useful in confirming the presence of obstruction or perforation. Dye studies to confirm the origin and communication of the fistulae are required especially in the setting of Crohn's disease. Intra-luminal dye studies are generally avoided in the acute setting as they are time consuming and serve little purpose. In our case, the obvious fecal material exuding from the fistulous tract and the presence of pneumoperitoneum warranted exploration. The increasing use of computed tomography scans in the surgical emergency can with some accuracy identify the loop of bowel that has herniated into the inguinal canal and give information on the condition of the strangulated contents. The presence of bowel wall edema and non-enhancement of the bowel wall following intravenous contrast portend a gangrenous bowel.

Most of the spontaneous entero-scrotal fecal fistulae have been reported from resource poor countries like Nigeria,[6–8] Pakistan,[5] and India.[2] Such fistulae have been reported after laparoscopic inguinal hernia repair as well.[10] This report is unique because a true sliding hernia presenting as a scrotal fecal fistula is not reported in literature, this probably being the first case in an adult.

## CONCLUSION

This report highlights the need for an early diagnosis followed by immediate surgical management of inguinal hernia. Standards of care have improved in countries like India yet such complications are not uncommon. Native indigenous treatment of inguinal swellings in the rural areas, poverty, and ignorance on the part of the patient from seeking proper medical care are the main causes for such presentation. Spontaneous entero-scrotal fistula is a rare presentation in the adult and reflects the state of health care in the developing countries. Timely elective surgery saves the patient not only from such complications but also saves money spent on treatment and less loss of working days.
